# Real-world comparison of bleeding risks among non-valvular atrial fibrillation patients prescribed apixaban, dabigatran, or rivaroxaban

**DOI:** 10.1371/journal.pone.0205989

**Published:** 2018-11-01

**Authors:** Ping G. Tepper, Jack Mardekian, Cristina Masseria, Hemant Phatak, Shital Kamble, Younos Abdulsattar, William Petkun, Gregory Y. H. Lip

**Affiliations:** 1 Department of Epidemiology, University of Pittsburgh, Pittsburgh, Pennsylvania, United States of America; 2 Department of Outcomes & Evidence, Pfizer, Inc; New York, New York, United States of America; 3 Department of Global Health Economics and Outcomes Research, Bristol-Myers Squibb; Princeton, New Jersey, United States of America; 4 Department of Global Medical Affairs, Bristol-Myers Squibb; Princeton, New Jersey, United States of America; 5 Institute of Cardiovascular Sciences, University of Birmingham, City Hospital, Birmingham, United Kingdom; Institut d'Investigacions Biomediques de Barcelona, SPAIN

## Abstract

Limited real-world data are available regarding the comparative safety of non-vitamin K antagonist oral anticoagulants (NOACs). The objective of this retrospective claims observational cohort study was to compare the risk of bleeding among non-valvular atrial fibrillation (NVAF) patients prescribed apixaban, dabigatran, or rivaroxaban. NVAF patients aged ≥18 years with a 1-year baseline period were included if they were new initiators of NOACs or switched from warfarin to a NOAC. Cox proportional hazards modelling was used to estimate the adjusted hazard ratios of any bleeding, clinically relevant non-major (CRNM) bleeding, and major inpatient bleeding within 6 months of treatment initiation for rivaroxaban and dabigatran compared to apixaban. Among 60,227 eligible patients, 8,785 were prescribed apixaban, 20,963 dabigatran, and 30,529 rivaroxaban. Compared to dabigatran or rivaroxaban patients, apixaban patients were more likely to have greater proportions of baseline comorbidities and higher CHA_2_DS_2_-VASc and HAS-BLED scores. After adjusting for baseline clinical and demographic characteristics, patients prescribed rivaroxaban were more likely to experience any bleeding (HR: 1.35, 95% confidence interval [CI]: 1.26–1.45), CRNM bleeding (HR: 1.38, 95% CI: 1.27–1.49), and major inpatient bleeding (HR: 1.43, 95% CI: 1.17–1.74), compared to patients prescribed apixaban. Dabigatran patients had similar bleeding risks as apixaban patients. In conclusion, NVAF patients treated with rivaroxaban appeared to have an increased risk of any bleeding, CRNM bleeding, and major inpatient bleeding, compared to apixaban patients. There was no significant difference in any bleeding, CRNM bleeding, or inpatient major bleeding risks between patients treated with dabigatran and apixaban.

## Introduction

Atrial fibrillation (AF) increases the risk of stroke and systemic embolism, and AF-related strokes have higher mortality, disability, costs, and risk of recurrent stroke compared to non-AF related strokes [1,2]. Oral anticoagulation with warfarin reduces the risk of stroke by 64%, and all-cause mortality by 26%, compared to control or placebo [3]. However, interactions with food and other drugs, variability in metabolism, a delayed onset of action, and the necessity of regular anticoagulation monitoring are limitations of warfarin therapy as well as a significant risk of major bleeding, particularly if anticoagulation control is poorly managed [4–6]. One population-based cohort study reported a major bleeding rate of 3.8% per person-year over a 5-year follow-up period [7]. This increased risk of bleeding with warfarin may lead to more discontinuations of oral anticoagulants, thus exposing patients to a risk of stroke and mortality.

Currently, non-vitamin K antagonist oral anticoagulants (NOACs) offer relative efficacy, safety, and convenience compared to warfarin. These drugs can be given in fixed doses without routine coagulation monitoring, and they have minimal drug and food interactions [7,8]. In clinical trials, NOACs were non-inferior or superior to warfarin for the prevention of stroke or systemic embolism in moderate-to-high risk patients with non-valvular AF, and were also non-inferior or superior to warfarin in terms of safety, with regard to major and intracranial bleeding [9]. However, clinical trials are limited by strict inclusion/exclusion criteria, and the generalizability to everyday clinical practice requires post-licensing ‘real world’ observational studies.

With the recent licensing and availability of NOACs, including dabigatran etexilate mesylate, rivaroxaban, apixaban, and edoxaban, data are needed on their comparative safety profile in many countries. Dabigatran was approved in the United States in 2010, while rivaroxaban, apixaban, and edoxaban were approved in 2011, 2012, and 2015, respectively.

The objective of this retrospective claims observational cohort study was to compare the risk of bleeding among non-valvular atrial fibrillation (NVAF) patients prescribed apixaban, dabigatran, or rivaroxaban.

## Materials and methods

This is a retrospective observational cohort study using insurance claims data from the Truven MarketScan Commercial Claims and Encounter and Medicare Supplemental & Coordination of Benefits Early View Database incurred from 01JAN2013-31OCT2014 to capture the real-world experience of NVAF patients who were either new initiators or switchers from warfarin. The database captures person-specific clinical utilization among approximately 100 payers of large employers, health plans, and government and public organizations in the United States, with more than 196 million unique patients since 1995. The database included annual insurance claims of inpatient, outpatient, emergency room, pharmacy, behavioural health care, and enrollment data for more than 94 million insured individuals, their dependents for active employees, early retirees, Consolidated Omnibus Budget Reconciliation Act (COBRA) health plan continuers, and Medicare-eligible retirees with employer-sponsored private health insurance and employer-provided Medicare Supplemental plans in the United States [10]. Data extraction for the purpose of this study was compliant with the Health Insurance Portability and Accountability Act (HIPPA).

The study population consisted of patients with an AF diagnosis claim (N = 1,209,729) during the study period. Patients were identified based on at least 1 inpatient or 2 outpatient claims that were at least 30 days apart, with a primary or secondary diagnosis of AF (International Classification of Diseases, Ninth Revision, Clinical Modification [ICD-9-CM]: 427.3). The first AF diagnosis claim during the study period was defined as the date of AF diagnosis for this population. As documented in claims data, we excluded transient perioperative AF patients and patients with valvular heart disease or hyperthyroidism at the time of AF diagnosis and women who were pregnant during the study period. Transient perioperative AF patients were identified as patients who had cardiac surgery procedures (ICD-9-CM: 35–39) up to 30 days before the AF diagnosis date. Valvular heart disease was identified based on inpatient or outpatient diagnosis of mitral stenosis or prosthetic heart valve (ICD-9-CM: 394, 396, 424, or 746). Hyperthyroidism was defined as having an inpatient or outpatient diagnosis of hyperthyroidism or thyrotoxicosis (ICD-9-CM: 242).

**Fig 1:**
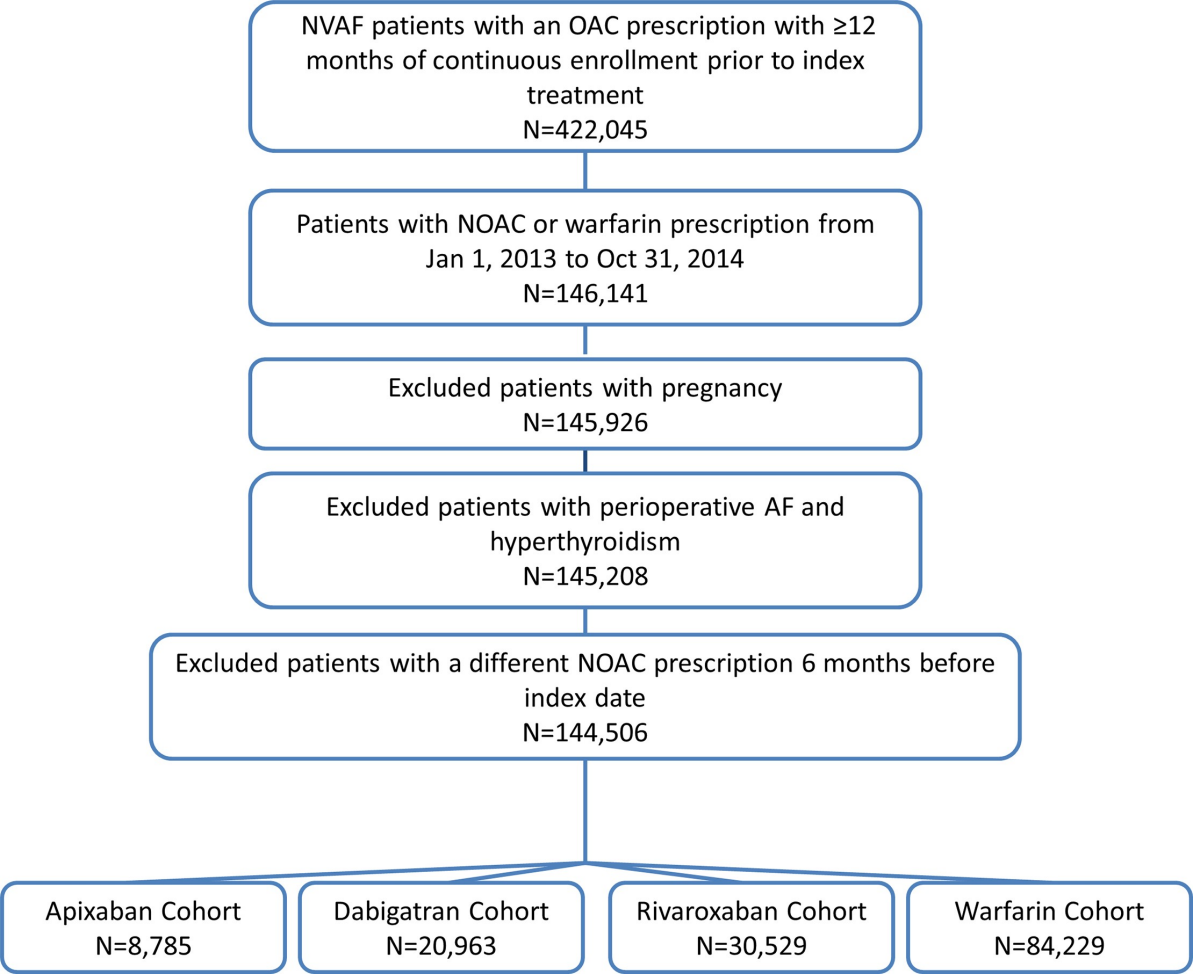
Patient selection criteria. AF: atrial fibrillation; NOAC: non-vitamin K antagonist oral anticoagulant; NVAF: non-valvular atrial fibrillation; OAC: oral anticoagulant.

NVAF patients who had unique pharmacy claims for apixaban, dabigatran, or rivaroxaban on or after their AF diagnosis date were identified (n = 146,141) from 01JAN2013-31OCT2014. The date of the first prescription claim was identified as the index date. The population included new initiators of unique NOACs and those who switched from warfarin. Allowing warfarin experienced patients in the study population makes it more representative of ‘real-world’ practice. All patients had 12 months of continuous enrollment prior to their index date. Patients with bleeding, stroke, or transient ischemic attack (TIA) within 30 days prior to or on the index date were excluded to avoid ambiguity about timing of treatment initiation and occurrence of events. Patients who had a different NOAC prescription 6 months before the index date were excluded (Fig 1).

Follow-up started after the index date and ended with the occurrence of bleeding, health plan disenrollment, discontinuation, switch of therapy, or 6 months after treatment initiation, whichever came first. Discontinuation of therapy was defined as no evidence of index prescriptions for 30 days from the last day of supply of the last filled prescription. The date of discontinuation was the last day of supply of the last filled prescription. During follow-up, if the NOAC initiator had a pharmacy claim for another NOAC, the patient was censored on the first date of the new drug’s pharmacy claim.

Any bleeding, including major and clinically relevant non-major (CRNM) bleeding, was defined using inpatient or outpatient claims with a primary diagnosis of bleeding. Inpatient major bleeding was identified based on inpatient claims, with major bleeding as the primary diagnosis for a hospitalization (any visit to a hospital for haemorrhage). The definition of major bleeding was modified from a published administrative claims-based algorithm and captures major bleeding at key sites including but not limited to intracranial, gastrointestinal (GI), liver, splenic, and ocular hemorrhage requiring hospitalization with a diagnosis for bleeding [11]. Inpatient major bleeding was further categorized into intracranial haemorrhage (ICH), GI, and other bleeding. The ICD-9-CM codes used to identify bleeding are listed in the Supplementary Material.

Baseline patient characteristics during the 12-month period before or on the index date were determined. Demographic factors included age on the index date, sex, health plan type, and geographic region. Baseline comorbidities were identified based on inpatient or outpatient claims with diagnoses of interest. Clinical prediction risk scores, including Charlson Comorbidity Index (CCI), CHADS_2_ and CHA_2_DS_2_-VASc stroke risk, and HAS-BLED bleed risk scores were calculated as allowed by the availability of the data [12–15]. The CHADS_2_ risk index was based on a point system in which 2 points are assigned for a history of stroke or a transit ischemic attack and 1 point each is assigned to age≥75 years, a history of hypertension, a history of diabetes mellitus, or a heart failure. CHADS2-VASc score was calculated with further consideration for vascular disease. The system will include 1 point for congestive heart failure, hypertension, diabetes mellitus, vascular disease (prior myocardial infarction [MI], peripheral artery disease, or aortic plaque), aged 65–74, and female, and 2 points for age ≥75, stroke/TIA/thromboembolic disease. Modified HAS-BLED score was calculated to approximate bleed risk. One point was assigned to patients with 1) hypertension (ideally systolic blood pressure >160 mm hg, but for this study, ICD-9 code was used), 2) abnormal renal function, 3) abnormal liver function, 4) stroke, 5) history of bleeding or predisposition (anemia), 6) elderly (aged >65 years), 7) concomitant antiplatelet or nonsteroidal anti-inflammatory drugs, and 8) alcohol abuse [15].

Prior stroke and bleeding in the baseline period were also reported. Concomitant use of antiplatelets, nonsteroidal anti-inflammatory drugs (NSAIDs), angiotensin-converting-enzyme (ACE) inhibitors, statins, and other anticoagulants 120 days preceding or on the index date were identified based on pharmacy claims. Patients who switched from warfarin to an NOAC were identified. Index NOAC dosage was categorized as reduced (apixaban 2.5 mg twice a day; dabigatran 75 mg twice a day; rivaroxaban 15 mg once a day), standard (apixaban 5 mg twice a day; dabigatran 150 mg twice a day; rivaroxaban 20 mg once a day), or unknown.

### Statistical analysis

Descriptive statistics of patient characteristics were summarized as mean (Standard Deviation, SD), Median (interquartile range, IQR). Pairwise comparisons were conducted between dabigatran and apixaban as well as between rivaroxaban and apixaban using Pearson’s chi-square test and the Kruskal-Wallis test for categorical and continuous variables, respectively. Overall annualized rates of inpatient bleeding were calculated for the first 6 months. Time-to-bleeding was modelled using Cox proportional hazard regression. Multivariate modeling was performed with the adjustment of baseline risk factors including age, gender, baseline comorbidities, and medications. Risk of bleeding, when comparing dabigatran or rivaroxaban versus apixaban, was expressed as adjusted hazard ratios (HRs) and 95% confidence intervals (CIs). Statistical significance was determined using 2-sided tests with alpha = 0.05 and reported as p-values <0.001 (***), <0.01 (**), <0.05 (*)

Two sensitivity analyses were conducted. First, a sensitivity analysis was conducted using only patients who received the standard dosage (apixaban 5 mg twice a day; dabigatran 150 mg twice a day; rivaroxaban 20 mg once a day). Second, a sensitivity analysis based on inverse probability treatment weighting (IPTW) was performed. A multinomial logistic model with treatment group as response and covariates included in the Cox regression adjusted models was fit to calculate the weights. Weighted Cox proportional hazards models were used to estimate the time-to-inpatient major bleeding in the dabigatran and rivaroxaban cohorts compared with the apixaban cohort. All analyses were conducted using SAS Windows 9.3 (SAS Institute Inc., Cary, NC).

## Results

The eligible study population included 8,785 apixaban, 20,963 dabigatran, and 30,529 rivaroxaban patients. Of the 32,800 patients, the median follow-up duration was 184 days (interquartile range [IQR] 89–312) for apixaban, 553 days (IQR 341–619) for dabigatran, and 300 days (IQR 151–505) for rivaroxaban patients. The average age was 70 years for both apixaban and dabigatran patients and 68 years for rivaroxaban patients (Table 1). Clinical comorbidity profiles were more similar between apixaban and rivaroxaban patients than between apixaban and dabigatran patients. Apixaban patients had greater proportions of clinical comorbidities compared to both dabigatran and rivaroxaban patients, with higher overall CCI scores, higher stroke and bleeding risk scores, and greater use of antiplatelet drugs prior to the index medication; apixaban patients were more likely to have switched from warfarin (Table 1).

**Table 1:** Baseline characteristics of non-valvular atrial fibrillation (NVAF) patients who initiated apixaban, dabigatran, or rivaroxaban.

Patient Characteristics	Apixaban (n = 8,785) (Reference)	Dabigatran (n = 20,963)	Rivaroxaban (n = 30,529)
Age, Mean (SD), Median (IQR)	70 (12) 70 (61,80)	70 (11) 70 (61,79)	68 (12)*** 68 (60,78)
Aged ≥75, %	38.1	38.0	34.5***
Female, %	37.3	34.7***	36.8
Myocardial Infarction, %	7.1	5.0***	6.9
Peripheral vascular disease, %	8.8	7.4***	8.4
Congestive Heart Failure, %	19.0	17.3***	18.6
Diabetes mellitus, %	30.0	30.9	29.2
Renal Disease, %	10.8	8.5***	8.9***
Malignancy, %	12.1	11.3*	12.6
Hypertension, %	73.6	66.2***	69.2***
Anemia, %	3.6	2.6***	3.5
Alcohol Abuse, %	0.6	0.4**	0.7
Pulmonary Embolism, %	1.1	0.6***	4.5***
Deep Vein Thrombosis, %	0.9	0.6*	3.1***
Cardioversion, %	9.3	8.9	9.0
History of Bleeding	16.8	15.6**	18.3**
History of Stroke/ transient ischemic attack	5.8	3.8***	5.2*
**CHADS**_**2**_**, Mean (SD)**	**1.7 (1.1)**	1.6 (1.1)***	1.6 (1.1)***
0	12.8	14.5	15.6
1	32.5	34.3	34.0
2	32.8	33.3	30.8
3+	21.9	17.8	19.7
**CHA**_**2**_**DS**_**2**_**-VASc, Mean (SD)**	**2.5 (1.5)**	2.4 (1.4)***	2.4 (1.5)***
0	8.3	9.0	10.8
1	19.3	19.2	20.9
2	24.0	27.5	24.2
3+	48.5	44.3	44.1
**HAS-BLED, Mean (SD)**	**1.9 (1.2)**	1.8 (1.2)***	1.8 (1.2)***
0	9.6	10.9	7.5
1	30.0	32.7	28.1
2	35.3	35.3	36.3
3+	25.1	21.1	28.1
**CCI score, Mean (SD)**	**1.8 (2.0)**	1.6 (1.9)***	**1.8 (2.2)**
0	32.7	34.7	33.8
1	23.6	25.8	24.4
2	16.4	15.2	15.0
3+	27.3	24.4	26.9
Medication use 120 days preceding index dates, %			
Use of antiplatelets	9.3	4.2***	7.4***
Use of NSAIDs	7.1	12.3***	7.5
ACE inhibitors	32.7	33.4	31.3*
Antidepressants/antipsychotics	18.2	18.6	19.4*
Angiotensin receptor blockers	22.2	21.7	21.3
Statins	52.2	54.2**	48.3***
Other anticoagulants	1.5	0.9***	2.8***
Switched from warfarin, %	17.3	4.4***	15.7***
Dosage		***	***
Reduced	16.9	12.0	20.3
Standard	79.1	83.1	76.3
Unknown	4.0	4.9	3.4

***: p<0.001

**, p<0.01

*, p<0.05

ACE: angiotensin-converting-enzyme; CCI: Charlson Comorbidity Index; CHADS_2_: Congestive heart failure, Hypertension, Age ≥75 years, Diabetes mellitus, prior Stroke, transient ischemic attack or thromboembolism; CHA_2_DS_2_-VASc: Congestive heart failure, Hypertension, Age ≥75 years, Diabetes mellitus, prior Stroke or transient ischemic attack, Vascular disease, Age 65–74 years, Sex category; CHF: congestive heart failure; HAS-BLED: hypertension, Abnormal renal function, Abnormal liver function, previous Stroke, prior major Bleeding or predisposition, Labile international normalized ratio; Elderly age (>65 years), Drugs predisposing to bleeding, alcohol use; IQR: interquartile range; MI: myocardial infarction; NSAIDs: nonsteroidal anti-inflammatory drugs; NVAF: non-valvular atrial fibrillation; PVD: peripheral vascular disease; SD: standard deviation; TIA: transient ischemic attack

**Fig 2:**
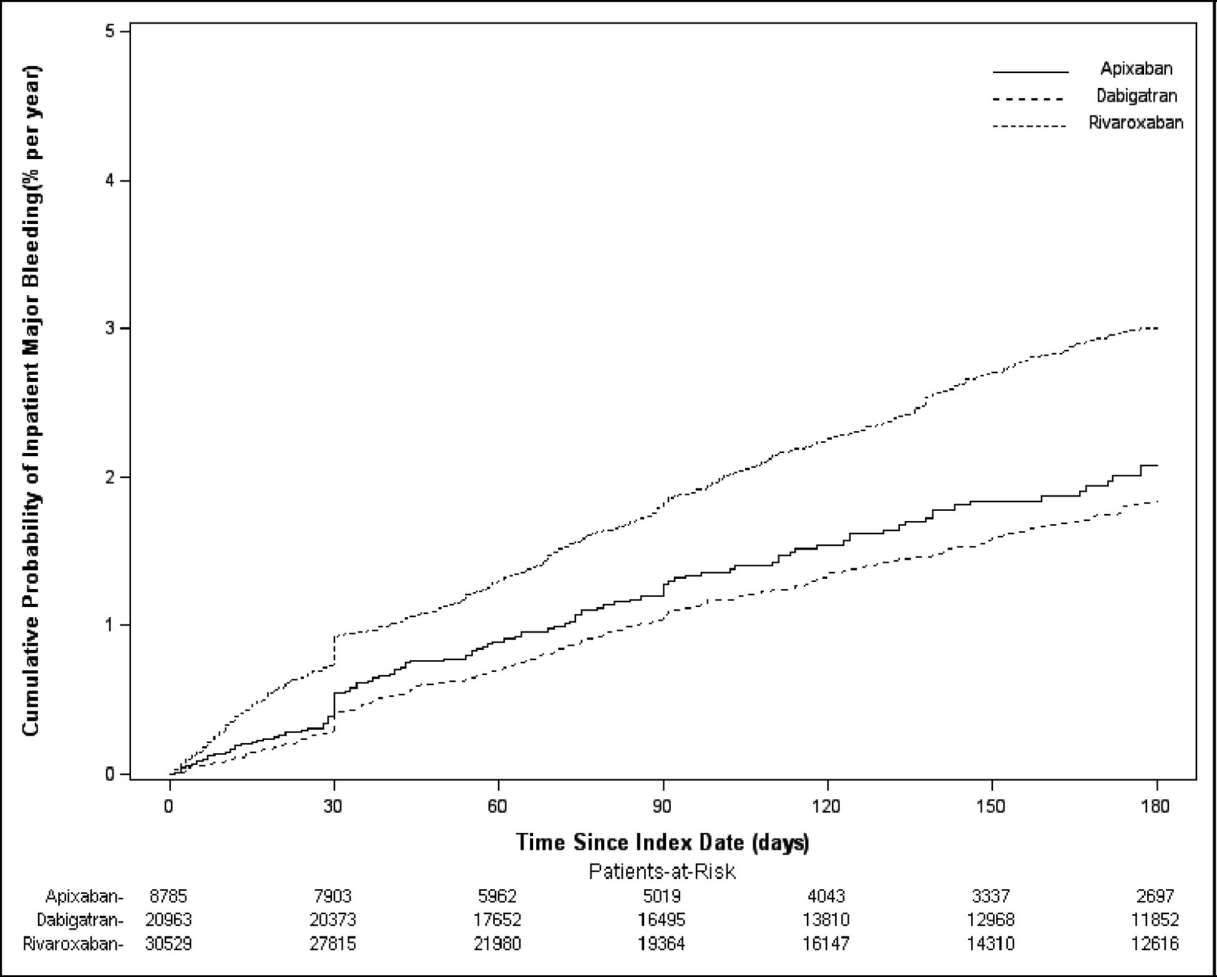
Kaplan-meier curves of any major inpatient bleeding by treatment. Rivaroxaban has the highest cumulative probability of any inpatient major bleeding. The overall Log-rank is p<0.0001.

The unadjusted bleeding rates are shown in Table 2, and the cumulative incidence of major bleeding is represented in Fig 2. After the adjustment of baseline patient characteristics–medication use, dosage, and switching from warfarin–patients treated with rivaroxaban were significantly more likely to have any bleeding (HR: 1.35, 95% CI: 1.26–1.45) or CRNM bleeding (HR: 1.38, 95% CI: 1.27–1.49) within 6 months of treatment initiation compared to those treated with apixaban (Table 3).

**Table 2:** Unadjusted annual cumulative incidence of bleeding among non-valvular atrial fibrillation (nvaf) patients who initiated apixaban, dabigatran, or rivaroxaban.

	Apixaban (N = 8,785)			Dabigatran (N = 20,963)			Rivaroxaban (N = 30,529)		
Bleeding	N	%	Incidence %/year	N	%	Incidence %/year	N	%	Incidence %/year
Any bleeding	962	11.0	39.5	2,828	13.5	37.7	4,855	16.0	53.5
Clinically relevant non-major bleeding	742	8.5	30.4	2,173	10.4	28.9	3,759	12.4	41.3
**Inpatient Major Bleeding**									
Total	119	1.4	4.6	306	1.5	4.9	656	2.1	6.7
Intracranial haemorrhage	13	0.1	0.5	36	0.2	0.5	64	0.2	0.7
Gastrointestinal	77	0.9	3.0	211	1.0	2.7	447	1.5	4.6
Other	40	0.5	1.5	94	0.4	1.2	251	0.8	2.6

CRNM: clinically relevant non-major (bleeding); GI: gastrointestinal; ICH: intracerebral haemorrhage; NVAF: non-valvular atrial fibrillation

**Table 3:** Adjusted hazard ratios (HRs) and 95% confidence intervals (CIs) for any, major, and clinically relevant Non-Major (CRNM) bleeding during the first 6 months after treatment initiation comparing dabigatran and rivaroxaban vs apixaban.

Bleeding	Adjusted HR (Dabigatran vs Apixaban)	P-value	Adjusted HR (Rivaroxaban vs Apixaban)	P-value
Any Bleeding	1.00 (0.93, 1.08)	0.88	**1.35 (1.32, 1.45)**	<0.0001
CRNM Bleeding	1.01 (0.93, 1.10)	0.83	**1.38 (1.27, 1.49)**	<0.0001

CI: confidence interval; CRNM: clinically relevant non-major (bleeding); HR: hazard ratio

After adjusting for baseline characteristics, there was a 43% (95% CI: 1.17–1.74) increased adjusted risk of inpatient major bleeding for rivaroxaban patients as compared to apixaban patients (Table 4). This effect was mainly observed in the risk of GI and other inpatient major bleeding with rivaroxaban as compared to apixaban, with a 51% (95% CI: 1.18–1.92) increased adjusted risk of GI inpatient bleeding, and a 58% (95% CI: 1.13–2.22) increased adjusted risk of other inpatient major bleeding.

**Table 4:** Adjusted hazard ratios (HRs) and 95% confidence intervals (CIs) for inpatient major bleeding during the first 6 months after treatment initiation comparing Dabigatran and Rivaroxaban vs Apixaban among non-valvular atrial fibrillation (NVAF) patients.

Inpatient Major Bleeding	Adjusted HR (Dabigatran vs Apixaban)	P-value	Adjusted HR (Rivaroxaban vs Apixaban)	P-value
Any	0.89 (0.72, 1.10)	0.29	**1.43 (1.17, 1.74)**	**<0.01**
Intracranial haemorrhage	0.95 (0.50, 1.80)	0.86	1.29 (0.71, 2.35)	0.41
Gastrointestinal	0.94 (0.72, 1.23)	0.67	**1.51 (1.18, 1.92)**	**<0.01**
Other	0.84 (0.58, 1.22)	0.35	**1.58 (1.13, 2.22)**	**<0.01**

CI: confidence interval; GI: gastrointestinal; HR: hazard ratio; ICH: intracerebral hemorrhage; NVAF: non-valvular atrial fibrillation

No significant differences were found between dabigatran and apixaban patients for any bleeding, CRNM bleeding, or inpatient major bleeding. The sensitivity analysis to assess the standard dose treatment effect on risk of major bleeding showed similar trends of significantly higher major risk with rivaroxaban compared to apixaban (Table 5). Additionally, the IPTW sensitivity analysis demonstrated consistent trends with the main analysis (Table 6).

**Table 5:** Sensitivity analysis using only patients initiated with standard dosage adjusted hazard ratios (HRs) and 95% confidence intervals (CIs) for inpatient major bleeding during the first 6 months after treatment initiation comparing dabigatran and rivaroxaban vs apixaban among non-valvular atrial fibrillation (NVAF) patients.

Major Inpatient Bleeding	Adjusted HR (Dabigatran vs Apixaban)	P-value	Adjusted HR (Rivaroxaban vs Apixaban)	P-value
Any	0.84 (0.67, 1.06)	0.14	**1.38 (1.11, 1.70)**	**<0.01**
Intracranial haemorrhage	1.00 (0.47, 2.14)	0.99	1.49 (0.73, 3.05)	0.27
Gastrointestinal	0.82 (0.62, 1.10)	0.19	**1.35 (1.04, 1.76)**	**0.03**
Other	0.86 (0.57, 1.30)	0.48	**1.52 (1.05, 2.21)**	**0.03**

CI: confidence interval; GI: gastrointestinal; HR: hazard ratio; ICH: intracerebral hemorrhage; NVAF: non-valvular atrial fibrillation

**Table 6:** Sensitivity analysis—Inverse probability treatment weighting IPTW analyses: Adjusted hazard ratios (HRs) and 95% confidence intervals (CIs) for inpatient major bleeding during the first 6 months after treatment initiation comparing dabigatran and rivaroxaban vs apixaban among nvaf patients.

Inpatient Major Bleeding	Adjusted HR (Dabigatran vs Apixaban)	P-value	Adjusted HR (Rivaroxaban vs Apixaban)	P-value
Any	1.02 (0.81, 1.28)	0.88	**1.54 (1.26, 1.89)**	**<0.01**
Intracranial haemorrhage	1.06 (0.55, 2.04)	0.87	1.45 (0.79, 2.66)	0.23
Gastrointestinal	1.10 (0.82, 1.47)	0.52	**1.65 (1.28, 2.11)**	**<0.01**
Other	0.98 (0.66, 1.46)	0.92	**1.69 (1.20, 2.38)**	**<0.01**

CI: confidence interval; GI: gastrointestinal; HR: hazard ratio; ICH: intracerebral hemorrhage; NVAF: non-valvular atrial fibrillation

## Discussion

In this study, our principal finding was that NVAF patients treated with rivaroxaban appeared to have an increased risk of any bleeding, CRNM bleeding, and inpatient major bleeding compared to patients treated with apixaban. There was no significant difference in any bleeding, CRNM bleeding, or inpatient major bleeding between dabigatran and apixaban patients.

This large observational cohort study compares inpatient bleeding risks among NVAF patients treated with the three NOACs: rivaroxaban, dabigatran, and apixaban. Despite greater comorbidities and worse bleeding and stroke profiles among apixaban patients, these patients experienced significantly less major inpatient bleeding, CRNM bleeding, or any bleeding events compared to rivaroxaban patients, and had comparable bleeding event rates to dabigatran patients. When compared with apixaban, rivaroxaban patients also showed significantly higher GI and other bleeding risks, and trended towards a higher ICH bleeding risk. Dabigatran had similar risks with apixaban across various bleeding sites.

Previous studies used data from large clinical trials to compare the safety between NOACs, which have been used to inform indirect comparisons and network meta-analyses [16]. Our study is broadly supportive of clinical trial observations, and in the ROCKET-AF trial, rivaroxaban had a comparable risk of bleeding to warfarin, whilst apixaban had significantly lower bleeding risk compared to warfarin [17–19]. We also found less bleeding with dabigatran compared to rivaroxaban, consistent with indirect comparison studies [20].

Few direct comparisons have been completed for apixaban, dabigatran, and rivaroxaban patients in a real-world setting. Another observational study using MarketScan data and propensity score matching showed that dabigatran had similar risk of major bleeding compared to apixaban and rivaroxaban, and apixaban had significantly lower risk of major bleeding compared to rivaroxaban [21]. Our study showed consistent results with additional comparisons of types of major bleeding and CRNM. Furthermore, in a more recent claims study using Optum claims data, apixaban patients had a 50% and 61% lower risk of major bleeding compared to dabigatran and rivaroxaban patients, respectively. There was no difference in the risk of ICH between apixaban and dabigatran or rivaroxaban patients [22]. In another study using the same data, apixaban patients also had a significantly lower risk of GI bleeding compared to dabigatran and rivaroxaban patients [23].

In addition, previous real-world studies have compared the risk of major bleeding for NOACs versus warfarin, the standard of care. Several real-world analysis comparing dabigatran to warfarin on adjusted overall bleeding risks showed greater or non-significant differences in overall bleeding, but higher GI bleeding and lower ICH risks [24–27]. Nonetheless, a recent study reported significantly lower overall major bleeding and ICH risks among dabigatran patients compared to warfarin patients [28]. Abraham et al. found similar GI bleeding risks when comparing dabigatran and rivaroxaban separately to warfarin using the Optum dataset [29]. Furthermore, real-world studies focused on rivaroxaban versus warfarin have shown no statistically significant difference in bleeding risk [21,28,30]. In addition, apixaban patients have been shown to have consistently lower risk of major bleeding compared to warfarin [21,28,31].

Based on large national claims data, our study adds novel evidence regarding the comparative bleeding risks of apixaban, dabigatran, and rivaroxaban in patients with NVAF. This population includes patients who were warfarin naïve and warfarin experienced, which makes it more representative of true clinical practice. Many prior studies only include treatment-naïve patients. Clearly, more real-world studies regarding bleeding risks and use of NOACs are still warranted.

### Limitations

First, health insurance databases include patients with varied risk profiles, and patients with a higher risk of major bleeding were more likely to use apixaban. Second, patients on all dosages of apixaban, dabigatran, and rivaroxaban were included in the study population. As expected, previous studies have shown that increased dosages are positively associated with bleeding events. Sensitivity analysis using only standard dosage found comparable results. Third, compared with clinical trials, no causal relation can be drawn in this retrospective cohort study. Additionally, there are wide ranges of comorbidities among the cohorts, and although baseline characteristics were adjusted, some residual confounding is likely because of unmeasured confounders [32]. The mean length of follow-up for patients treated with apixaban was significantly shorter compared to those treated with dabigatran and rivaroxaban. Survival methodology was used to account for the varied follow-up length; however, apixaban-related bleeding events could have occurred later than the other NOACs, which could have affected the results. Given the distinct separation in the cumulative incidence, we would expect minimal impact on the results.

Furthermore, there are inherent limitations of claim data, such as coding errors and missing data. Comorbidities were presented in the dataset using ICD-9-CM diagnosis codes. Laboratory data, including creatinine clearance, are not available in the claims database, so diagnosis codes were used to determine comorbidities. Additionally, with a claims database, medication as filled may not reflect true medication use [33]. Nonetheless, this study used a large database of nationally representative commercially insured patients and is one of the first studies to compare the safety between NOACs.

## Conclusions

In conclusion, NVAF patients treated with rivaroxaban appeared to have an increased risk of any bleeding, CRNM bleeding, and major inpatient bleeding compared to patients treated with apixaban. There was no significant difference in any bleeding, CRNM bleeding, or inpatient major bleeding between dabigatran and apixaban patients. These data may help guide decision-making in clinical practice.
